# Supplementary material for: A Global Screen for Assembly State Changes of the Mitotic Proteome by SEC-SWATH-MS
Source: Cell Syst. 2020 Feb 26;10(2):133–155.e6. doi: 10.1016/j.cels.2020.01.001 (PMC7042714; doi:10.1016/j.cels.2020.01.001)

P07237 | PDIA1_HUMAN | P4HB ERBA2L PDI PDIA1 PO4DB
Monomer MW [kDa]: 57.116 Monomer expected elution fraction: 45

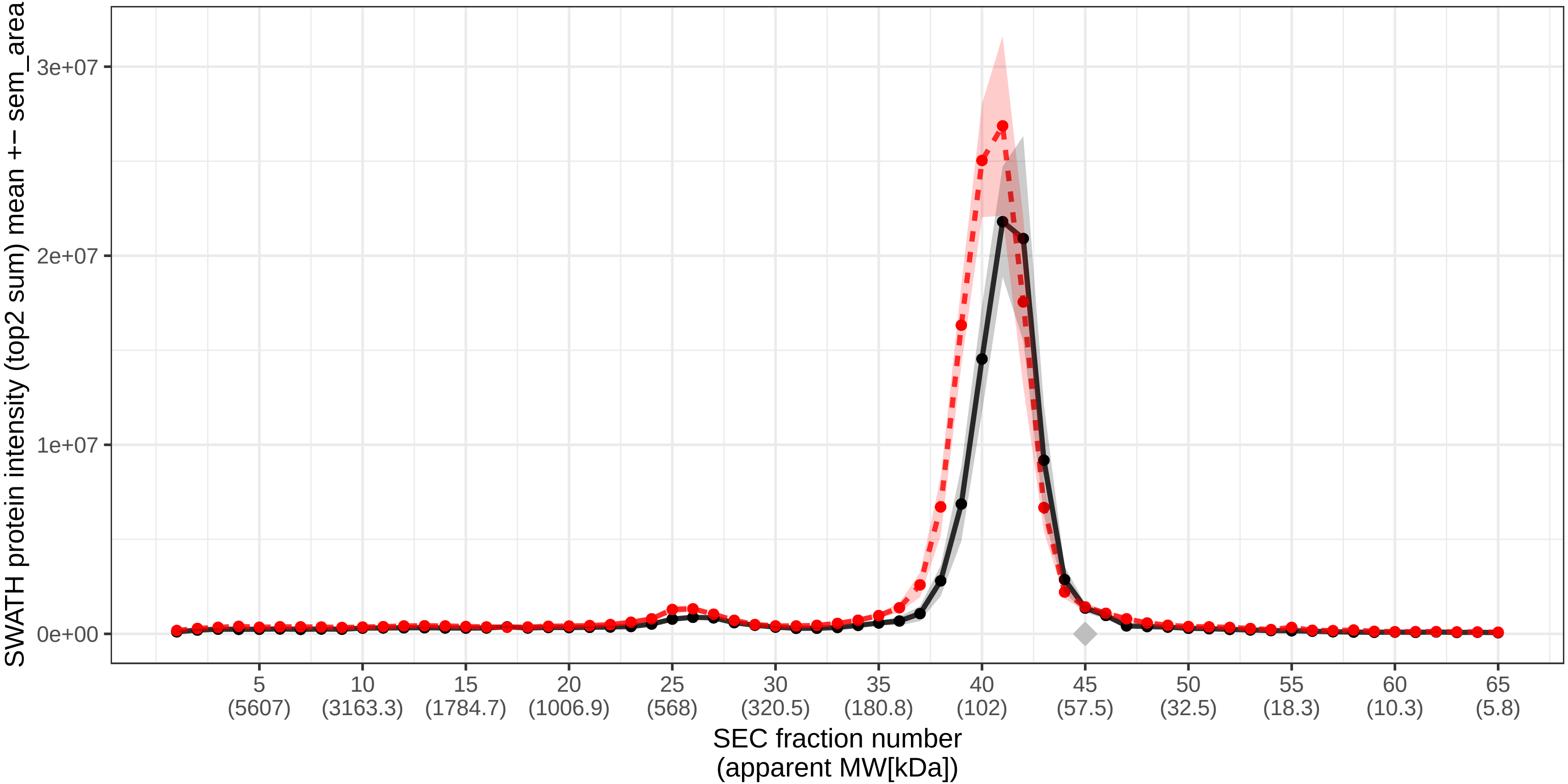

Supplement: Data S1. SEC-SWATH-MS Protein Chromatograms, Related to Figure 1 [file mmc6.zip › SECchrom_P07237_PDIA1_HUMAN_P4HB_ERBA2L_PDI_PDIA1_PO4DB.pdf]
